# The proGnostic role of caRdiac rehAbilitation in patients with left ventriCular anEurysm formation after anterior myocardial infarction (the GRACE study): Study rationale and design of a prospective randomized controlled trial

**DOI:** 10.3389/fcvm.2022.991521

**Published:** 2023-01-10

**Authors:** Peng Zhang, Xiaofeng Ge, Zhaokai Li, Meiling Nie, Jing Yu, Weimei Ou, Kaimin Wu, Jiahua Li, Lin Wang, Wei Ni, Zaixing Shi, Juan Song, Suiji Li, Cuilian Dai

**Affiliations:** ^1^Department of Cardiology, Xiamen Cardiovascular Hospital of Xiamen University, School of Medicine, Xiamen University, Xiamen, China; ^2^Xiamen Key Laboratory of Cardiovascular Disease, Xiamen Cardiovascular Hospital of Xiamen University, School of Medicine, Xiamen University, Xiamen, China; ^3^Key Laboratory of Health Technology Assessment of Fujian Province, School of Public Health, Xiamen University, Xiamen, China

**Keywords:** cardiac rehabilitation (CR), myocardial infarction, ventricular aneurysm, peak VO_2_, EuroQoL 5-Dimension (EQ-5D)

## Abstract

**Background:**

Cardiac rehabilitation (CR) is an essential intervention after acute myocardial infarction (MI). However, it is still unclear whether patients with left ventricular aneurysm (LVA) formation after anterior MI would benefit from CR programs. This clinical trial is designed to assess the role of CR in patients with LVA formation after anterior MI.

**Trial design:**

The GRACE study is a single-center, single-blind, prospective, randomized controlled clinical trial in China. 100 subjects aged 18–75 years with LVA formation after anterior MI will be recruited and randomized 1:1 to the CR or control group. Both groups will receive standard drug treatment and routine health education according to the guidelines. Participants in the CR group will additionally receive tailored CR programs delivered over a period of 36 sessions. These participants will then be followed up for 1-year. The primary outcome is peak oxygen uptake measured by cardiopulmonary exercise testing after CR programs. The secondary outcomes are cardiac function and EuroQol 5-Dimension-3 Level index scores after CR program and 1-year and major adverse cardiac cerebrovascular events, a composite of cardiovascular mortality, non-fatal MI, non-fatal stroke, malignant arrhythmia or hospitalization for heart failure during the follow-up period.

**Conclusions:**

This single-center, single-blind, prospective, randomized controlled clinical trial will determine whether CR improves physical capacity and clinical outcomes in patients with LVA formation after anterior MI.

**Trial registration:**

Chinese Clinical Trial Registry ChiCTR2200058852. Registered on 18 April 2022.

## Background

Acute myocardial infarction (MI) is a cardiac emergency with poor prognosis (1, 2). The mortality and morbidity of acute MI remain high despite the epidemiologic trend changes over the past decades. Mechanical complications are crucial determinants of the poor prognosis after MI (3).

True left ventricular aneurysm (LVA), defined as the abnormal outward of a dyskinetic or akinetic area of the left ventricular (LV) wall, is one of the most common mechanical complications of MI. This complication usually forms in the case of acute anterior MI without well-developed coronary collaterals (4). Within the first 2 weeks post-MI, cardiomyocyte death by apoptosis and necrosis occur and the collagen fibrils are disrupted in the infarct zone. Then the infarcted, non-contractile, myocardial tissue becomes expanded under the intraventricular pressure. Over time, the aneurysmal wall is replaced by fibrous tissue along with the remodeling of the myocardium, which results in decreased LV function (4, 5). Patients with LVA post-MI are at increased risk for heart failure, aneurysmal thrombosis or ventricular arrhythmias (6). In addition, LVA was associated with higher risk of cardiac mortality and complications (7). Therefore, it is necessary to improve the management of LVA after MI.

Exercise-based cardiac rehabilitation (CR) is a systematic, multidisciplinary approach to secondary prevention of cardiovascular diseases (8, 9). Exercise-based CR after acute MI is an integral component of care with Class I recommendation in American and European guidelines based on clinical and cost-effectiveness (10–12). Previous studies showed a positive prognostic value of CR in patients after MI (13, 14). A previous meta-analysis of 34 randomized trials has suggested that exercise-based CR was associated with reduced mortality and recurrent MI (15). However, it remains unclear whether patients with LVA formation after anterior MI could benefit from CR treatment. The change of peak oxygen uptake (peak VO_2_), the most well-established variable of cardiopulmonary exercise testing (CPET), will be measured as one of the primary outcomes before and after CR program in patients with LVA post-MI (16). Another primary outcome in this study is the health-related quality of life (HRQoL) measured by the EuroQol 5-Dimension-3 Level (EQ-5D-3L) after CR program and 1-year (17). This trial will also evaluate the effect of exercise-based CR on the change of cardiac function and the occurrence of major adverse cardiac cerebrovascular events (MACCE), a composite of cardiovascular mortality, non-fatal MI, non-fatal stroke, malignant arrhythmia or hospitalization for heart failure as the secondary outcomes during the follow-up period.

## Methods

### Study design

This is a single-center, prospective, randomized controlled, single-blind clinical trial. A total of 100 participants with LVA after acute anterior MI for at least 2 months will be enrolled from 1 May 2022 to 1 May 2024 and randomized (1:1) to the CR group or control group, receiving standard drug treatment and routine health education according to the guidelines. The diagnose of LVA depends on the transthoracic echocardiography. LVA was defined as a demarcated bulge of the contour of the left ventricular wall during both diastole and systole, which showed akinesia and dyskinesia (18). In cases with suboptimal acoustic window, contrast echocardiography will be used. All of the participants will undergo percutaneous coronary intervention (PCI) before enrollment.

**Figure 1:**
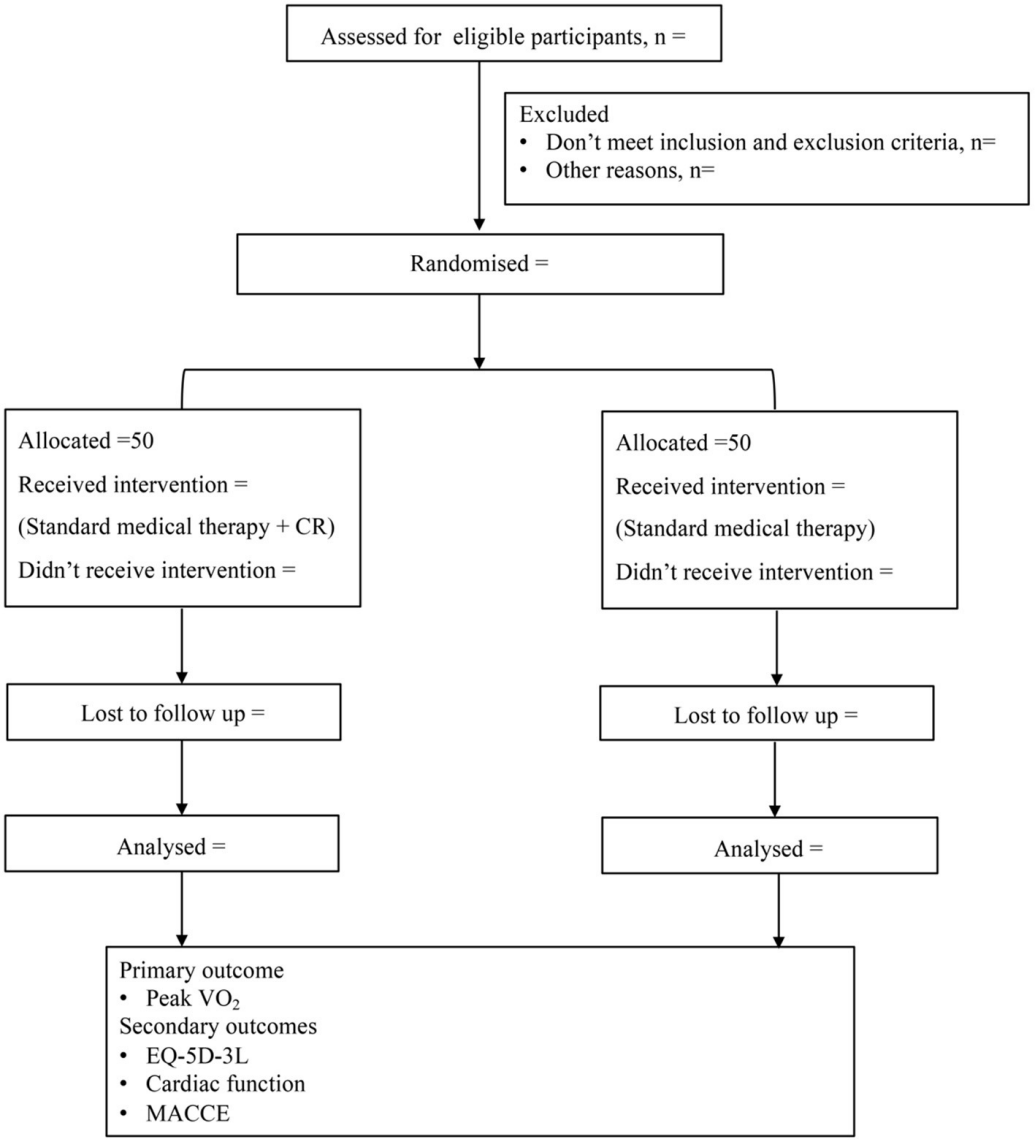
Study design. CR, cardiac rehabilitation; VO_2_, oxygen uptake; EQ-5D-3L, EuroQol 5-Dimension-3 level; MACCE, major adverse cardiac cerebrovascular events.

Participants in the CR group will receive physician-supervised home-based CR intervention. The CR program will be continued for 36 sessions. All of the participants will be followed up for 1-year. Cardiopulmonary exercise testing (CPET), transthoracic echocardiography and dynamic single-photon emission computed tomography (D-SPECT) will be performed before and after intervention. The incidence of MACCE will be recorded in the follow-up period according to the study protocol outlined in Figure 1.

The Standard Protocol Items: Recommendations for Intervention Trials (SPIRIT) statement will be followed in this trial (19). The study has been approved by the Ethics Committee of Xiamen Cardiovascular Hospital of Xiamen University (approved number: 2021YLK32) and registered at Chinese Clinical Trial Registry (registration number: ChiCTR2200058852).

### Participants

The trial will be conducted in Xiamen Cardiovascular Hospital of Xiamen University. Subjects aged 18–75 years, with LVA after acute anterior MI for at least 2 months are eligible if they are willing and able to complete the CR program. The eligible subjects will be screened according to the inclusion and exclusion criteria and determined by a cardiologist.

### Inclusion criteria

(1) Aged 18–75 years old.
(2) LVA Patients with anterior MI.
(3) Complete revascularization with PCI.

### Exclusion criteria

(1) LV thrombosis;
(2) Mechanical complications of acute myocardial infarction include LV free-wall rupture, ventricular septal rupture, mitral valve cord rupture or unstable hemodynamics;
(3) Malignant arrhythmia; decompensated heart failure; high-grade atrioventricular block; severe aortic valve stenosis; severe hypertrophic obstructive cardiomyopathy;
(4) Acute stroke; acute deep vein thrombosis or acute arterial thrombosis; acute asthmatic attack; malignant tumor; uncontrolled hyperthyroidism; acute renal failure; acute infection and severe motor dysfunction;
(5) Patients who require a surgical operation within the next 6 months which affects the continuous use of antiplatelet drugs;
(6) Participating in another clinical trial related to coronary interventional devices;
(7) Those who had an expected life span of <1 year;
(8) Inability or unwillingness to comply with the study requirements;
(9) Patients who dropped out during the study period.

### Sample size

The sample size of this study was generated using PASS (Power Analysis and Sample Size) version 15.0 statistical software (UT, USA) (20). Sample size calculations were conducted for two independent means. The parameters include predicted value of peak VO_2_, significance level (α) and power (1 – β). Based on previous study, the peak VO_2_ at baseline was 18.2 ± 4.4 ml/kg/min in control group and 18.8 ± 3.7 ml/kg/min in moderate-intensity CR group, the peak VO_2_ after exercise program was 17.1 ± 4.6 ml/kg/min in control group and 21.6 ± 4.5 ml/kg/min in moderate-intensity CR group (21). To achieve 90% power and a 2-sided critical threshold of 0.05, the sample size required in each arm is 37 subjects. Allowing for a drop-off rate of 25%, the recruitment goal is 50 subjects per arm (*n* = 100).

### Randomization and allocation concealment

Participants who are eligible and willing will be randomized into the CR group or the control group in a 1:1 ratio. Randomization was performed by an independent statistician using research randomizer (http://www.randomizer.org). Allocation of the participants to the CR group or control group will be done by sequentially numbered opaque sealed envelope (snose) method. The case numbers will be allocated to the participants and related to the random number in each group. The outcomes measures will be assessed by the study personnel who are blinded to the study arm to which participants are assigned.

### Intervention

All of the participants will receive standard drug treatment and routine health education according to the guidelines. Participants enrolled in the control group will not receive extra rehabilitation or physical activity. Participants enrolled in the CR group will receive tailored and progressive CR intervention. The CR program consists of tailored in-hospital training sessions for 3 times and home-based CR program for 36 sessions with supervised sessions per week (22). The CR prescription will be made by physical therapist according to the results of CPET.

Tailored in-hospital CR intervention will be performed by experienced cardiac rehabilitation physicians at the CR center in the Xiamen Cardiovascular Hospital of Xiamen University. Evaluation will be conducted before each session. Participant will enter the exercise session according to the random number if not presented with uncontrolled cardiac arrhythmias, hemodynamic disturbances; acute decompensated heart failure within 24 h, second-degree or complete atrioventricular block, acute renal failure, hyperthyroidism; infection or motor system dysfunction. After evaluation, participants will receive personalized moderate-intensity exercise (50% peak VO_2_) based on the results of CPET. The exercise intensity could be properly adjusted according to rate of perceived exertion (RPE) scale during the rehabilitation program. In general, RPE between 11 and 13 on the Borg Scale is the intended target. It will take 30 min to complete the training cycle per session. The training cycle includes a 5-min warm-up period, followed by the 20-min aerobic exercise and a 5-min recovery period. Safety during the training cycle will be ensured by monitoring blood pressure (BP), oxygen saturation (SaO_2_), heart rate (HR) and electrocardiogram (ECG). Indications for terminating exercise testing are as follows: symptoms including chest pain, shortness of breath, wheezing, cyanosis, pallor, fatigue, ataxia, dizziness, syncope, leg cramps, claudication or any other unbearable discomfort; ST-segment elevation >1.0 mm or horizontal or down-sloping ST segment depression >2 mm; arrhythmia including ventricular fibrillation, sustained ventricular tachycardia, R-on-T ventricular premature beat, frequent multifocal ventricular premature, supraventricular tachycardia, and bradyarrhythmias or exercise-induced bundle-branch block that cannot be distinguished from ventricular tachycardia; drop in systolic BP > 10 mmHg from baseline despite an increase in workload accompanied by any other evidence of ischemia; hypertensive response (systolic BP > 220 mm Hg and/or diastolic BP > 110 mm Hg); subject's request to stop.

After tailored and progressive in-hospital training sessions for 3 times, participants will continue to perform physician-supervised home-based exercises 5 sessions per week and 30 min per session, scheduled for 36 sessions. The home-based exercise consists of moderate intensity aerobic exercise at a target HR reserve (HRR). Participants will be required to perform outdoor walking exercise at an intensity of 50% of HRR based on the results of CPET monitored by a pulse-watch. The required exercise intensity will be measured subjectively using a Borg score of 11–13. Details about the benefits of home-based CR program in optimizing cardiovascular health will be provided to the participants. A booklet is encouraged to use to record exercise time, HR and any discomfort during exercise. Participants will receive telephonic feedback on training frequency, duration and intensity from the physical therapist per week during the 36 sessions. The physical therapist will give feedback on training parameters measured during the preceding week and discuss the progress with respect to the personal training goals. Then, the participants will be advised to continue their training with the HR monitor. Participants will receive in-hospital training guidance every 2 weeks. A long-term home-based exercise plan will be made by the physical therapist after the CR program. Subjects will be followed up for 12 months.

### Outcomes

The primary outcome of this study is peak VO_2_ after CR program since no definitive evidence of the impact of CR on peak VO_2_ in post-MI patients with LVA has been found so far. One of the secondary outcomes of this study is HRQoL at after CR program and 1-year quantified with the EQ-5D-3L index scores, which comprises five dimensions including mobility, self-care, usual activities, pain/discomfort, and anxiety/depression. Each dimension of the EQ-5D-3L has three levels: no problems, some problems, and severe problems, resulting in 243 kinds of different health states (23, 24). Other secondary outcomes of this study include the change of cardiac function after CR program and 1-year and the occurrence of MACCE at 1-year.

In addition, other indicators of CPET such as VO_2max_, anaerobic threshold (AT), VO_2/_HR, VO_2_/kg, carbon dioxide production (VCO_2_), ventilatory equivalent (VE), VE/VO_2_, VE/CO_2_, VE/CO_2_ slope, Metabolic equivalents (METs), HR reserve (HRR), HR_max_, VO_2_/HR, BP, ΔVO_2_/ΔWR, respiratory quotient (RQ), breathing reserve (BR), tidal volume (VT), VT/inspiratory capacity (IC), dead space volume (VD)/VT, arterial partial pressure of oxygen (PaO_2_), P (A-a) O_2_, PaCO_2_, end-tidal partial pressure of CO_2_ (PET CO_2_) and P (a-ET) CO_2_ will be obtained before and after CR program.

The cardiac function indicators by transthoracic echocardiogram and D-SPECT are as follows: LV end diastolic volume (LVEDV), LV end systolic volume (LVESV), LV end diastolic dimension (LVEDD), LV ejection fraction (LVEF), interventricular septum (IVST). In transthoracic echocardiography, LVEF was estimated using the biplane modified Simpson's method (18). Myocardial perfusion will be evaluated by D-SPECT and the following indicators will be studied: summed thickening score (STS), summed motion score (SMS), summed stress score (SSS), summed rest score (SRS), summed difference score (SDS), total perfusion defect (TPD), summed thickening score (STS), and hibernating myocardium (HM). Additionally, soluble source of tumorigenicity 2 (sST2), a myocardial fibrosis biomarker, will be detected before and after CR program as exploratory outcome (25). The outcomes will be ascertained independently and blindly by Clinical Events Committee. A diagram timeline of the study is presented in Table 1.

**Table 1:** The study timeline.

	**Baseline**	**After CR program**	**At 12 months**
Demographics	X		
Medication	X	X	X
Physical examination	X	X	X
Laboratory examination	X	X	X
Echocardiography	X	X	X
sST2	X	X	
CPET	X	X	
EQ-5D-3L	X	X	X
D-SPECT	X	X	
MACCE	X	X	X

CR, cardiac rehabilitation; sST2, soluble source of tumorigenicity; CPET, cardiopulmonary exercise testing; EQ-5D-3L, EuroQol 5-Dimension-3 level; D-SPECT, dynamic single-photon emission computed tomography; MACCE, major adverse cardiac cerebrovascular events.

### Data management

The database will be established by EpiData version (Atlanta, Georgia, USA) based on the CRFs project. Relevant original information will be extracted independently by the researchers. The study data will be monitored by researchers at the School of Public Health of Xiamen University every 3 months.

### Data analysis

Normally distributed continuous variables are expressed as mean ± SD and performed with paired or unpaired Student's *t*-test. Non-normally distributed continuous variables are recorded as median with interquartile range (IQR) and determined by Wilcoxon signed-rank test or Mann-Whitney *U* test. Categorical variables are presented as frequency and percentage (*N*, %) and evaluated by χ^2^ test or Fisher's exact test as appropriate. Kaplan-Meier analysis are used to calculate the mean survival time of the patients. An interim analysis is added during the trial. Both the intention to treat (ITT) principle and the per protocol (PP) analyses will be presented in our study. *P* < 0.05 (two-sided) is considered to be statistically significant. Statistical analysis will be conducted using SPSS 25.0 statistical software (IBM, Chicago, IL, USA).

## Discussion

LVA, one of the fatal complications of MI, is resulted from myocardial necrosis and fibrosis and usually lead to LVEF reduction and cardiac death (5). Although the early coronary revascularization and the use of anti-remodeling drugs can help to improve prognosis in patients with LVA after MI, further exploration of cardiac secondary prevention interventions are needed. Considering this, patients with LVA after anterior MI are taken as the target population in this study.

The significance of CR in MI has been highlighted by the American Heart Association and European Society of Cardiology in recent years (11, 12). Evidence from basic science showed CR post MI could attenuate cardiac remodeling and improve cardiac function through increase microcirculation, eliminate free radicals and regulate autonomic dysfunction (26). Additionally, CR has beneficial role in improving quality of life and cardiorespiratory fitness in patients after MI. Home- and center-based forms of CR have been proven to be similar in improving clinical and HRQoL outcomes in patients after MI (22). However, little attention has been paid to the impact of CR on the LVA formation after MI. The current study will address the role of CR on cardiac function and clinical outcomes in post-MI patients with LVA.

Peak VO_2_, the highest value of oxygen uptake obtained during the exercise, is used to estimate the cardiorespiratory fitness (27). The benefits of CR are mediated mainly by improving cardiorespiratory fitness. Previous study showed that each increase of 1 ml·kg^−1^·min^−1^ in Peak VO_2_ was associated with a 15% decrease in mortality (28). Peak VO_2_ is considered to be an independent predictor for long-term survival in patients with coronary artery disease (29). The GRACE study directly targets the change of peak VO_2_ before and after personalized moderate intensity CR program and we hypothesize that the exercise training will significantly increase peak VO_2_ in participants with LVA after MI. HRQoL is becoming an important outcome measure in MI due to the high mortality and morbidity rates from MI. The potential benefits of CR post-MI on HRQoL has been recognized recently (30). EQ-5D-3L questionnaire is widely applied to evaluate HRQoL and has been validated in patients with MI (17, 30). In this study, we hypothesize that the EQ-5D-3L index scores will be increased after moderate-intensity CR treatment in participants with LVA formation after anterior MI. This study will also shed light on the change of cardiac function, the occurrence of MACCE and levels of sST2 in post-MI patients with LVA. This study will therefore contribute to the existing knowledge on the prognostic impact of CR in patients with LVA after anterior MI.

## Conclusions

In summary, the GRACE study is a prospective randomized controlled trial that will provide evidence of whether CR is safe and effective for patients with LVA after myocardial infarction. Home-based CR may be a reasonable option for stable post-MI patients with LVA who are eligible for CR but not convenient to attend a traditional center-based CR program.

## Ethics statement

The studies involving human participants were reviewed and approved by the Ethics Committee of Xiamen Cardiovascular Hospital of Xiamen University. The patients/participants provided their written informed consent to participate in this study.

## Author contributions

JS, SL, and CD have conceived and designed the study. PZ, XG, ZL, JY, and MN will recruit and screen the participants. PZ, XG, ZL, WO, KW, JL, LW, and WN will participate in the data collection. PZ, XG, and ZL participated in drafting this manuscript. ZS, JS, SL, and CD provided the supervision support. All authors contributed to the critical revisions and final approval of the manuscript.
